# miR-155, miR-21, and let-7a Expressions in MCF-10A and MCF-7 Cell Lines after Low to High Dose Irradiation 

**DOI:** 10.22074/cellj.2021.7411

**Published:** 2021-10-30

**Authors:** Afsaneh Zare, Reza Fardid, Gholam Hossein Tamadon, Mohammad Amin Mosleh-Shirazi

**Affiliations:** 1. Department of Radiology, School of Paramedical Sciences, Shiraz University of Medical Sciences, Shiraz, Iran; 2. Ionizing and Non-Ionizing Radiation Protection Research Centre, School of Paramedical Sciences, Shiraz University of Medical Sciences, Shiraz, Iran; 3. Department of Laboratory Sciences, School of Paramedical Sciences, Shiraz University of Medical Sciences, Shiraz, Iran; 4. Diagnostic Laboratory Sciences and Technology Research Centre, School of Paramedical Sciences, Shiraz University of Medical Sciences, Shiraz, Iran; 5. Physics Unit, Department of Radio-Oncology, School of Medicine, Shiraz University of Medical Sciences, Shiraz, Iran

**Keywords:** Breast Cancer, Ionizing Radiation, Let-7a, MiR-155, MiR-21

## Abstract

**Objective:**

Ionizing radiation is a tremendous risk factor for cancer development. MicroRNAs (miRNAs) are regulators that utilize cell pathways, which are implicated in human cancer prognosis. In addition, miRNAs respond to anti-cancer therapy and proliferation after irradiation. However, the changes in miRNA expression profiles in response to irradiation have not been comprehensively analysed. The present study was designed to assess potential changes that occur in miRNA expression following irradiation.

**Materials and Methods:**

In this experimental study, we used quantitative real-time polymerase chain reaction (qRT- PCR) to measure the expressions of miR-155, miR-21, and let-7a in MCF-10A (normal breast cells) and MCF-7 (breast cancer cells) six hours after the cells were exposed to five different irradiation doses (50, 100, 400, 2000, and 4000 mGY).

**Results:**

After irradiation from the low to high doses, we observed an upsurge in miR-155 (more than 100%) expression and reduction in let-7a (more than 87%) expression. However, there was an increase and a reduction in miR-21 expression (more than 100%).

**Conclusion:**

Irradiation can play an important role in cancer development in normal breast cells (MCF-10A) at low dose irradiation. However, the results showed little difference at high doses of radiation.

## Introduction

Breast cancer is a complicated disease with genetic diversity that encompasses a wide range of changes in structure and gene expression. Since breast cancer is very prevalent and is the second cause of mortality amongst women, further research is vital in this field (1).

MicroRNAs (miRNAs) play important role in the diagnosis and treatment of cancers (2). MiRNAs comprise 19-25 nucleotides and they play a role in the arrangement of gene expressions. There are a few studies on radiation and miRNAs (3). In addition, miRNAs play a major role in several processes, such as apoptosis, differentiation, cell migration, and proliferation (4). Recently, the number of identified miRNAs in the process of gene arrangement has significantly increased (3). In order to achieve their intended target, miRNAs can play the role of tumour suppressor or oncogene (5).

MiRNAs can be potential candidates for breast cancer biomarker and used for early prognosis and diagnosis (6). MiR-155 increases in several cancers, including breast cancer (7). Recently, the influence of evidence-based miR-155 on suppressor of cytokine signalling 1 (SOCS1) was found to play a role in tumour suppression. An increase in miR-155 results in a decrease in SOCS1 levels (8). In addition, in another study the impact of miR-155 on caspase3 was observed as a suppressor with a major role in apoptosis (9).

In a study of rat breast cancer epithelial cells, miR-155, an inter-mediator in the TGFB pathway, was found to have a central role in cell formation epithelial-mesenchymal transition (EMT) and increased considerably (10). In general, although miR-155 increases in breast cancer, in some research, a decrease in miR-155 in some hormonal receptors has been seen (11). Increases in miR-21 have been reported in some cancers, including breast cancer (12, 13). The increase in miR-21 in cancer cells was shown to be specific when compared to normal breast cells (13). An investigation of 199 breast cancer patient and 21 healthy control by real-time polymerase chain reaction (qRT-PCR) showed a significant level of miR-21 (14). The amount of miR-21 expression in grades 2 and 3 cancer cells is higher than grade 1. MiR-21 is one of the major regulators of miRNAs in different cell pathways; this miRNA regulates metastasis and can control cell viability. Tropomyosin-1 is an important growth-inhibiting protein that is negatively regulated by miR-21 (15-17). 

Let-7a is part of the first known miRNAs, with a 12 member’s family (18). Let-7a regulates many targets in cell pathways and the levels of these targets increase in breast cancer. There is a negative feedback between let-7a and lin-28 (19). Gene producer let-7a is on chromosome 13, a fragile part of the gene that is constantly being deleted. Studies have shown that in undifferentiated cells, let-7a is also absent and the chance of cancer is also increased (20). The results of one study showed a decrease in let-7a in breast cancer cells (BT-IC) and an increase in let-7a in differentiated cells (21). Dingo and colleagues reported a role for let-7a in breast cancer metastasis by RKIP regulation. RKIP is a gene suppressor in breast cancer cells (22). The changes in miRNA expression profiles in response to irradiation have not been comprehensively analyzed. The present study was designed to assess potential changes that occur in miRNA expression following irradiation.

## Materials and Methods

This experimental research was designed to examine the effect of ionizing radiation on expression changes in three miRNAs - miR-155, miR-21, and let-7a. This study was conducted at Shiraz University of Medical Sciences with the support of the Vice Chancellor for Research and was registered by the Ethics Committee (IR.SUMS. REC.1397.538).

### Cell culture

We obtained both human breast cancer cell lines (MCF-7) and human normal breast cell line (MCF-10A) from Pasteur institute of Iran (Tehran, Iran). The cells were supplemented with 10% foetal bovine serum (FBS, Merck, Germany) and 1% penicillin-streptomycin (Merck, Germany), and cultivated in 20 flasks in medium at 37˚C and 5% CO_2_ under carefully controlled conditions. Each group was cultured in three flasks and twice testing was performed for each flask.

### Ionizing radiation treatment

The cultured cells were irradiated with different doses of ionizing radiation. The cells received 50, 100, 400, 2000, and 4000 mGY administered by a linak accelerator (Elekta Company, Sweden) at 6 mv and source-skin distance (SSD) of 100 cm and a dose rate of 200 mu/minutes. The experiments were conducted at Namazi Hospital, Shiraz, Iran. The irradiated cells were maintained at 37˚C and 5% CO_2_ for six hours prior to total RNA extraction conducted in accordance with the manufacturer’s instructions. 

### RNA extraction

Total RNA was extracted from the cultivated MCF-7 and MCF-10A cell lines using TRIzol reagent (Invitrogen, Carlsbad, CA, USA) in accordance with the manufacturer’s instructions. Both the RNA concentration and integrity were quantified in each sample by a NanoDrop (Bioner, South Korea) instrument before the samples were stored at -70˚C. 

### cDNA synthesis and real-time polymerase chain reaction

cDNA synthesis was conducted with a KIT protocol (Exiqon, Denmark) according to the manufacturer’s instructions. An average of 2 µl of the total miRNAs was used for cDNA synthesis based on the evaluation of the NanoDrop instrument. For recognizing miRNAs expression and RT-PCR, we used 10 ml of ROX and SYBR-green (Exiqon, Denmark), 1 µl cDNA, 1 µl primer, and 8 µl DEPC Water (DW) according to the manufacturer’s instructions. In order to specify the amount of miRNA expression and for RT-PCR analysis, we used Oligo-dT primer to design specific primers for miR-155, MiR-21, and let-7a. MiR-5s was purchased from mentioned company (Exiqon, Denmark), for internal control. The expression levels of these three miRNAs were evaluated by an ABI Step One QPCR according to the manufacturer’s instructions. Briefly, all samples were incubated for 60 minutes at 42˚C, followed by heat inactivation of the reverse transcriptase (RT-enzyme) at 95˚C for 5 minutes. Additionally, 5S rRNA gene was used as an internal control. The RT-PCR reactions were performed at 95˚C for 10 minutes, followed by 40 cycles at 95˚C for 10 seconds, and 60˚C for one minute with an ABI 7500 qRT-PCR system (Applied Biosystems, Foster City, CA, USA).

### Bioinformatics analysis

Identification of putative and validated target genes among the differentially expressed genes for all the studied seven miRNAs was performed by using Web-based software analysis. The corresponding gene, miRBase ID, and sequence of each miRNA in this study were assigned before analysis. The Web-based software used to investigate the miRNA targets were: miRTarBase (http://miRtarbase.mbc. nctu.edu.tw), miRecords (http://miRecords.biolead.org/), TargetScan (http://www.targetscan.org/), miRanda (http:// www.microrna.org), DIANA microT (http://diana.imis. athena-innovation.gr/DianaTools), and miRwalk (http://zmf. umm.uni-heidelberg.de/apps/zmf/miRwalk2/).

### Statistical analysis 

All experiments were performed in duplicate. Data were analysed by software used in an ABI instrument by taking into consideration the obtained cycle threshold (CT) numbers and the estimated melting curve using device for any miRNA in each irradiation. We had a CT number which was normalized to internal control (miR-5s) by Graph Pad Prism 5.0 (Graph Pad Software, Inc., La Jolla, CA, USA), finally use 2 ^(ΔΔ ct)^ were expressed as log2 values. 

## Results

**Fig.1:**
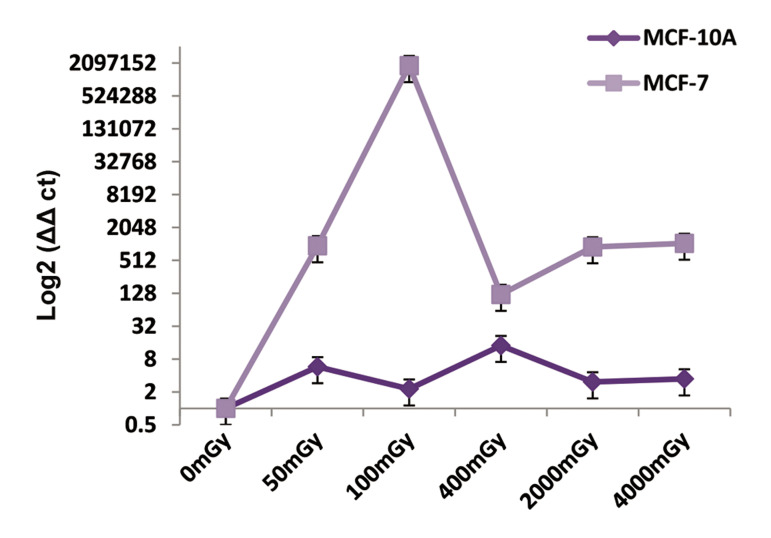
The relative expression of miR-155 in the MCF-10A and MCF-7 cell lines after irradiation at 50, 100, 400, 2000, and 4000 mGY. MicroRNA (miRNA) expressions were assessed after six hours. There is a chance for breast cancer in the low to high doses.

The MCF-7 and MCF-10A cell lines were cultivated under appropriate conditions and subsequently irradiated with varying doses (50, 100, 400, 2000, or 4000 mGY). After six hours, we evaluated miRNA expression levels by qRT-PCR. Irradiation from low to high doses showed an increase in miR-155 expression in the cancer cell line in comparison with normal cells, and this represented the probability of breast cancer at the various doses. Down-regulation occurred between the 100 to 400 mGY doses, which could be considered a reduction related to the chance of breast cancer at the 400 mGY dose compared to the lower doses. We observed up-regulation in the doses after 400 mGY (Fig .1)

**Fig.2:**
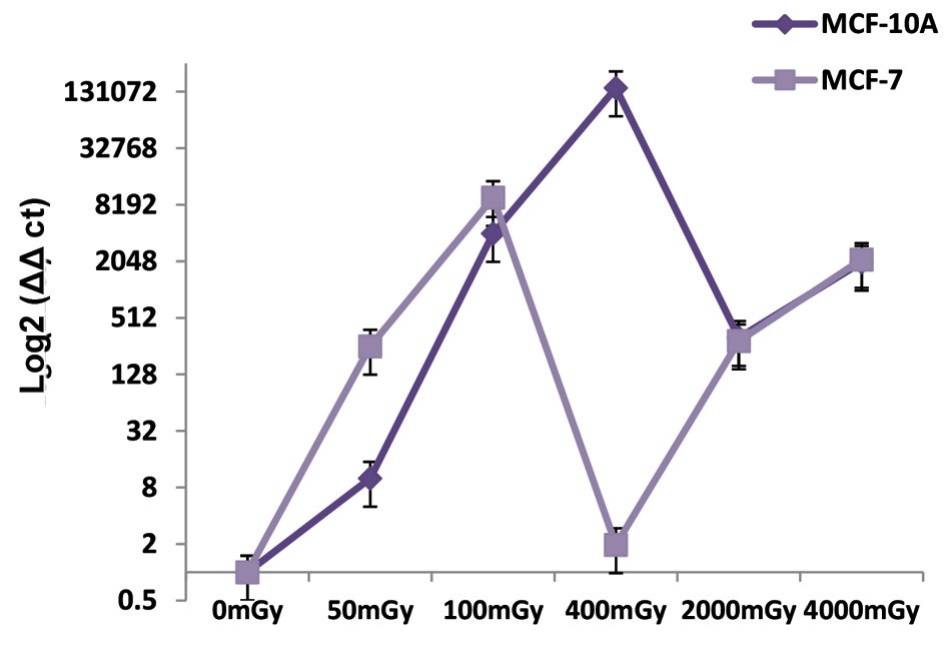
Relative expression of miR-21 in the MCF-10A and MCF-7 cell lines after irradiation at 50, 100, 400, 2000, and 4000 mGY. MicroRNA (miRNA) expressions were assessed after six hours. Up-regulation and down-regulation were observed at different doses.

MiR-21 expression was up-regulated and down-regulated in the cancer cells in comparison with the normal cells. There was up-regulation in 50 and 100 mGY doses; hence, it could be said that the probability of breast cancer increased in this dose due to the increase in miR-21 expression in breast cancer. However, we had a decrease in the expression of miR-21 in the cancer cells compared to normal cells at the 200 mGY dose, followed by an overlap to 4000 mGY. The minimum possibility to develop cancer in miR-21 after irradiation was 400 mGY where we had a significant difference between normal cells and cancer cells (Fig .2).

**Fig.3:**
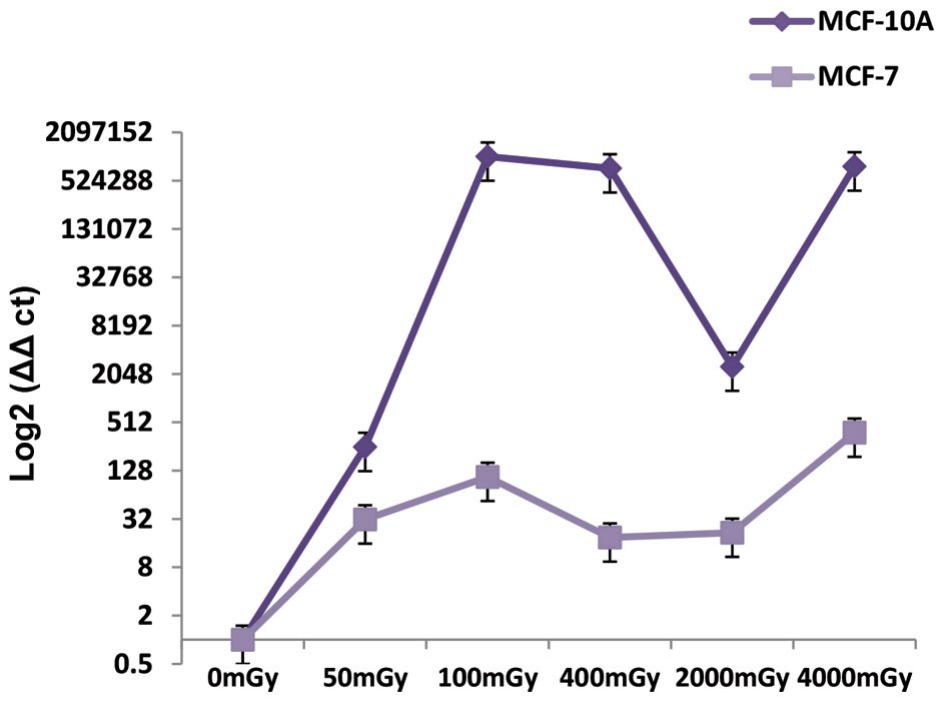
Relative expression of let-7a in the MCF-10A and MCF-7 cell lines after irradiation at 50, 100, 400, 2000, and 4000 mGY. MicroRNA (miRNA) expression was assessed after six hours. There is a chance of breast cancer in the low dose to high doses.

The reduction in let-7a in cancer cells compared to normal cells is a fact. Overally, we observed a decrease in let-7a expression in cancer cells compared with normal ones, which could be considered as an increased chance for breast cancer in the low to high doses. There was no significant increase or decrease in let-7a expression in the low to high doses; however, the most significant amount change to level of let-7a expression between normal and cancer cells was observed at the 400 mGY dose (Fig .3).

**Fig.4:**
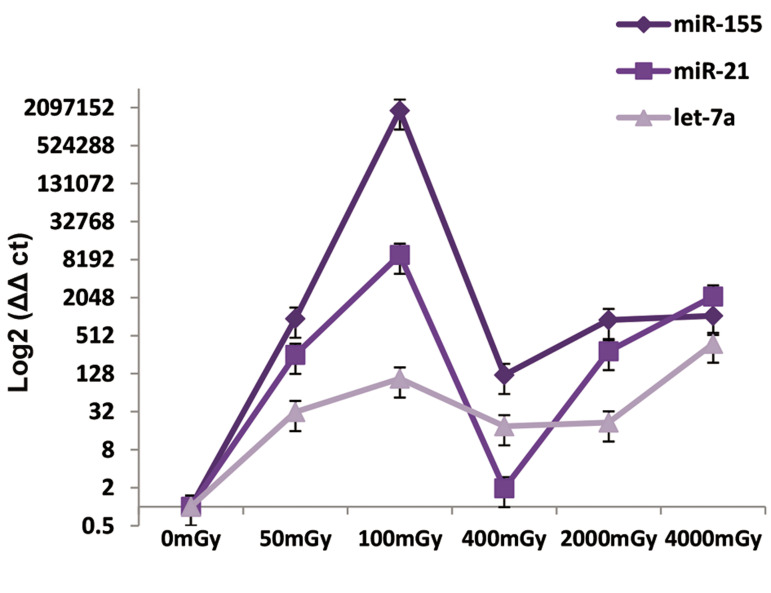
Relative expressions of miR-155, miR-21, and let-7a in the MCF-7 cell line after irradiation at 50, 100, 400, 2000 and 4000 x-rays. MicroRNA (miRNA) expression was assessed after six hours.

The MCF-7 cancer cell line was affected by let-7a down-regulation and miR-155 and miR-21 up-regulation. In terms of the irradiation effect, we observed a similarity in expressions of miR-155 and miR-21 in the cancer cells after irradiation. The higher probability of breast cancer was observed at the 100 mGY dose and the minimum probability was at the 400 mGY dose. For let-7a, the most significant effect was observed with the least possible expression, which occurred with the 400 mGY dose and the least irradiation effect was at 4000 mGY, which had the most expression (Fig .4).

**Fig.5:**
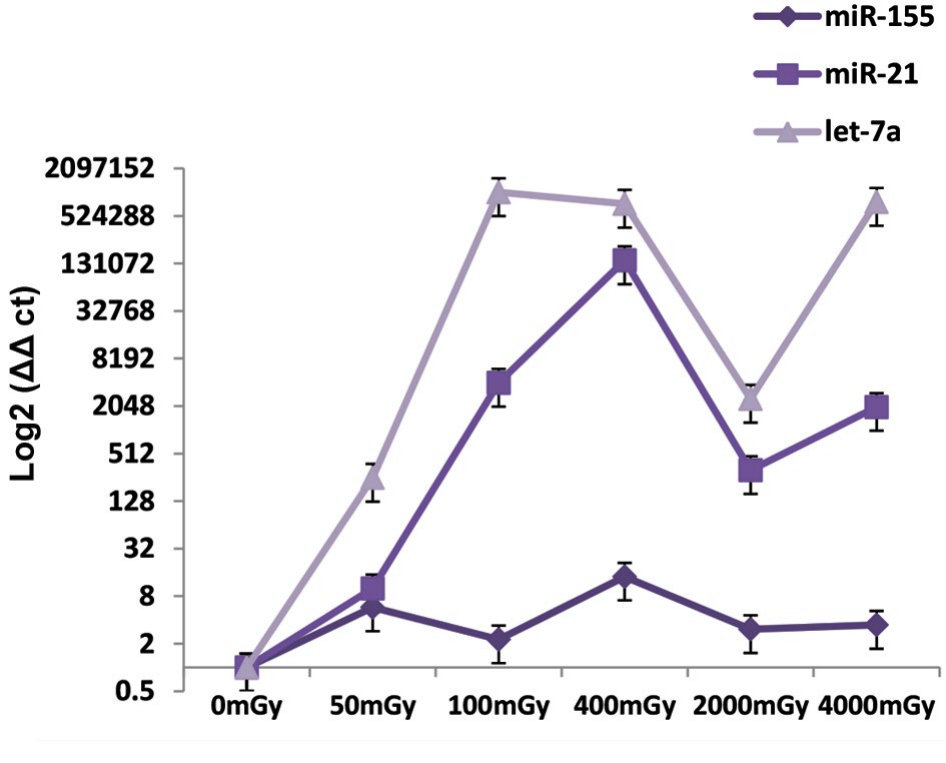
Relative expressions of miR-155, miR-21, and let-7a in the MCF-10A cell line after irradiation at 50, 100, 400, 2000, and 4000 mGY. MicroRNA (miRNA) expression was assessed after six hours.

The changes in expressions of miR-155, miR-21 and let-7a in the MCF-10A cell line (normal cells) are seen in the form of increase and decrease on different doses. In the case of let-7a, probability of breast cancer increased at the 2000 mGY dose compared to the 4000 mGY dose, due to a reduction in expression level. MiR-21 expression in normal cells at different doses showed that the probability of breast cancer has increased followed by a decrease at 2000 mGY compared to 400 mGY. MiR-155 expression in normal cells had no significant decrease or increase; however, it can be said that probability of breast cancer after irradiation was high at the 400 mGY dose compared to the other doses because we observed the highest expression (Fig .5).

## Discussion

There are recent significant developments in the field of molecular analysis and problem-solving aetiology. MiRNAs are a new category of endogenous RNA molecules that have aroused great interest in the scientific community. MiRNAs are often expressed in cancer. A new source of upstream molecular factors of gene expression has been discovered, which warrants extensive research to clarify their roles in cancer.

We studied the association between miRNAs and breast cancer. The role of miRNAs as an effective diagnostic factor for breast cancer was the focus of our research. MiRNAs have important roles in cancer diagnosis that are very important like germ cell tumours (23). Although one of the most important factors that influences the process of cancer is radiation, there are few studies of irradiation on miRNAs and breast cancer. A recent study showed the effect of different radiation doses on two types of cancer cell lines through the miRNA pathway (24). miRNAs are also used to treat breast cancer (25).

A double strand break (DSB) is a fatal damage caused by ionizing radiation that should be restored; one of the major components in this restoration is RAD51.

Gasparini et al. (26) reported that the increase in miR-155 in breast cancer cells would lead to a reduction in RAD51 levels and would affect the cell response to radiation. Consequently, along with radiation and an increase in miR-155 and consequent decrease in RAD51, cell sensitivity to radiation will increase. The results of the present study indicated increased miR-155 expression in the normal cell line compared to the cancer cell line, which could increase radio-sensitivity and the chance of breast cancer. Although in different doses we observed an increase and decrease. An important finding of miR-155 is the relationship with BRCA1. Chang et al. (27) showed that BRCA1 play an important role in damage to DNA repair and cell cycle. The function of BRCA1 will decrease with cell mutations and increases in miR-155. Based on the results of our research, an increase in miR-155 expression and a decrease in the performance of BRCA1 can lead to an increased chance for breast cancer.

One direct target of miR-21 is CDC25a, which has a tremendous effect on cell repair and increase of checkpoint arrest (28, 29). Also, miR-21 can be use as a radio-resistance, since, with increasing radiation dose and the need for DNA repair, an increase in miR-21 and CDC25a has been observed and this result can be seen in the 100 mGY dose. Although miR-21 is observed in breast cancer, there is no clear pattern of gene target of this miRNA, like PTEN; therefore, additional investigation is necessary (15) .

Let-7a has a key role in proliferation, differentiation and tumour suppression (30). There are contradictory results regarding increases or decreases in let-7a after radiation. The results of a study on lung cancer indicated down-regulation of let-7a. There was up-regulation in the entire let-7a family in a glioma cell line (31). In addition, the p53 path in determining the amount of let-7a expression is interesting after radiation. In a study after ionizing radiation (IR), we observed down-regulation of let-7a in cells sensitive to radiation, such as the lungs and bone marrow, while there was up-regulation in let-7a in cells resistant to radiation, like the brain and muscles (32). This change could come from several factors, including the type and amount of radiation and paths involved in let-7a regulations (e.g., line 28 and RAS) (33). We observed a significant decrease in let-7a in the cancer cells compared to the normal cells; hence, it can be inferred that there was a chance of breast cancer from the low dose to the high dose of irradiation.

## Conclusion

According to the results from various studied that investigated the relationship between miRNAs and radiation, we concluded that irradiation affects breast and other cancers. Our findings showed up-regulation of miR-155 and let-7a with an increase in irradiation dose. It could be said that the probability of breast cancer increases following irradiation in normal and cancer cells. However, we did not see miR-21 up-regulation with increased irradiation. We observed up-regulation and down-regulation at different doses. We could not say that the probability of breast cancer increased after irradiation in miR-21. The current and previous research studies could be a promising approach for the effect of radiation on miRNA expression. The role of miRNAs in breast cancer is suggested in diagnostic radiology and radiotherapy and in radiation accidents. 
